# Optimization and Evaluation of the In Vitro Permeation Parameters of Topical Products with Non-Steroidal Anti-Inflammatory Drugs through Strat-M^®^ Membrane

**DOI:** 10.3390/pharmaceutics13081305

**Published:** 2021-08-20

**Authors:** Bartłomiej Milanowski, Hanna Wosicka-Frąckowiak, Eliza Główka, Małgorzata Sosnowska, Stanisław Woźny, Filip Stachowiak, Angelika Suchenek, Dariusz Wilkowski

**Affiliations:** 1 Chair and Department of Pharmaceutical Technology, Poznan University of Medical Sciences, ul. Grunwaldzka 6, 60-780 Poznań, Poland; hwosicka@ump.edu.pl (H.W.-F.); eglowka@ump.edu.pl (E.G.); 2 GENERICA Pharmaceutical Lab, Regionalne Centrum Zdrowia Sp. z o. o., ul. Na Kępie 3, 64-360 Zbąszyń, Poland; m.sosnowska@rcz-zbaszyn.pl (M.S.); s.wozny@rcz-zbaszyn.pl (S.W.); f.stachowiak@rcz-zbaszyn.pl (F.S.); 3 MYLAN Healthcare Sp. z o. o., ul. Postępu 21B, 02-676 Warszawa, Poland; angelika.suchenek@viatris.com (A.S.); dariusz.wilkowski@viatris.com (D.W.)

**Keywords:** Strat-M^®^ membrane, in vitro permeation test, Plackett–Burman design, etofenamate, diclofenac, ibuprofen, ketoprofen, naproxen

## Abstract

Pharmaceutical products containing non-steroidal anti-inflammatory drugs (NSAIDs) are among the most prescribed topical formulations used for analgesic and antirheumatic properties. These drugs must overcome the skin barrier to cause a therapeutic effect. Human skin has been widely used as a model to study in vitro drug diffusion and permeation, however, it suffers from many limitations. Therefore, to perform in vitro permeation test (IVPT), we used a Strat-M^®^ membrane with diffusion characteristics well-correlated to human skin. This study’s objective was to optimize the IVPT conditions using Plackett–Burman experimental design for bio-predictive evaluation of the in vitro permeation rates of five non-steroidal anti-inflammatory drugs (diclofenac, etofenamate, ibuprofen, ketoprofen, naproxen) across Strat-M^®^ membrane from commercial topical formulations. The Plackett–Burman factorial design was used to screen the effect of seven factors in eight runs with one additional center point. This tool allowed us to set the sensitive and discriminative IVPT final conditions that can appropriately characterize the NSAIDs formulations. The permeation rate of etofenamate (ETF) across the Strat-M^®^ membrane was 1.7–14.8 times faster than other NSAIDs from selected semisolids but 1.6 times slower than the ETF spray formulation.

## 1. Introduction

Non-steroidal anti-inflammatory drugs (NSAIDs), such as diclofenac diethylamine (DEA) or sodium (DNa), etofenamate (ETF), ibuprofen (IBP), ketoprofen (KTP), and naproxen (NPX), are the most popular drugs used in topical formulations for their analgesic, antirheumatic, and anti-inflammatory properties. NSAIDs are recommended in international and national guidelines as an early treatment option for symptomatic management of knee and hand osteoarthritis. They may be used ahead of oral NSAIDs due to their superior safety profile [1].

Topical pharmaceutical formulations, designed to permeate the skin, require in vitro release-rate testing (IVRT) to produce reliable and consistent results. The release of active pharmaceutical ingredient (API) from all batches of the produced preparation should proceed at the same rate, and deviations from the norm will indicate manufacturing errors. Therefore, according to the Food and Drug Administration (FDA) [2] and the United States Pharmacopeia (USP) [3] guidelines, it is necessary to compare the release rates of API from semisolid forms to confirm the identical quality of two batches of the product after introducing changes in the composition or manufacturing process.

On the other hand, in vitro permeation test (IVPT) can help to explain and optimize the drug’s dermal absorption process during the development phase of a semisolid pharmaceutical product. Human skin has been widely used as a model for studying ex vivo diffusion of transdermal and topical formulations [4,5]. However, human skin suffers from high biological variabilities such as thickness, hair follicles density, lipid content, and composition [4,6,7]. Its usage is also limited by its availability, high cost, and special storage requirements [6,7,8]. What is more, excised skin may be prone to artifacts brought on by storage conditions, as some studies clearly show that skin freezing (especially below −20 °C) results in increased permeation of tested APIs [9].

Strat-M^®^ membrane (Merck Millipore, Burlington, MA, USA) was introduced as a synthetic membrane for in vitro diffusion studies as a substitute for human skin [10,11], however it cannot replace it. Strat-M^®^ membrane is composed of tight top layer (resembling stratum corneum), two layers of polyethersulfone (resembling dermis), lying atop one layer of polyolefin, which is more open and diffusive (playing the role of subcutaneous tissue). These multiple layers of the membrane create a general structure similar to that of human skin. In addition, this membrane is characterized by its low batch-to-batch variability, safety, and lack of storage limitations, thus providing more consistent data. Besides, it has been shown that the diffusion data of Strat-M^®^ membrane correlate well with those of human skin [7,10,11,12]. These findings suggest that Strat-M^®^ membrane can be used as an alternative to animal or human skin during in vitro permeation/diffusion studies, being a screening tool for evaluating topical/transdermal formulations [13,14,15].

The IVPT optimization and validation approach had not been meaningfully advanced until the Ng et al. work [16]. The parameters investigated included Franz cell’s dimensions, stirring conditions, membrane type, membrane treatment, temperature regulation, and sampling frequency. It was determined that validation of the optimized method dramatically reduced data variability as the coefficient of variation for steady-state ibuprofen permeation from a gel formulation was reduced from 25.7% to 5.3% (*n* = 6).

To the best of the authors’ knowledge, no systematic development of the IVPT of NSAIDs through Strat-M^®^ membrane using the Design of Experiments (DoE) approach has been published so far. One type of experimental design used for the optimization of the analytical method is the Plackett–Burman design [17,18,19,20]. This efficient two-level fraction factorial screening design identifies the statistically significant independent variables (factors) influencing response with very few experimental runs, which results in saving of chemicals, time, and human resources. The most important aspect of this design is combining different independent variables with a variable level (i.e., −1 and +1). Through Plackett–Burman design, a maximum of N-1 independent variables can be examined in N runs, where N is a multiple of 4. In this way, seven factors can be tested within eight runs, so the number of trials may be reduced to an absolute minimum. The Plackett–Burman design analyses the input data and presents a rank order of the variables with a magnitude of an effect and designates signs to the effects to indicate whether an increase in factor values is advantageous or not. Thus, the objective of this work was to:

1. Develop and validate a single Ultra High Performance Liquid Chromatography (UHPLC) assay applicable to quantify all five NSAIDs (DNa/DEA, ETF, IBU, KTP, NPX) used in the study since we have no found such a method during an extensive literature search;
2. Optimize the IVPT parameters using an automatic set of vertical Franz diffusion cells and Plackett–Burman factorial design to obtain bio-predictive IVPT based on ETF and DEA permeation across Strat-M^®^ membrane;
3. Evaluate NSAIDs’ in vitro permeation parameters (i.a. fluxes) from selected topical products obtained under optimized IVPT conditions.

## 2. Materials and Methods

### 2.1. Materials

Nine different commercially available formulations of NSAIDs for topical administration were purchased from a local pharmacy and used in this study: Dolgit^®^ cream 5% (manufacturer: DOLORGIET GmbH & Co. KG, Bonn, Germany), Ketonal^®^ gel 2.5% (manufacturer: Salutas Pharma GmbH, Sülzetal, Germany), Ketospray^®^ 10% (manufacturer: Pharbil Waltrop GmbH, Waltrop, Germany), Naproxen EMO gel 10% (manufacturer: EMO-FARM Sp. z o.o., Ksawerów, Poland), Olfen^®^ gel 1% (manufacturer: Merckle GmbH, Blaubeuren, Germany), Traumon^®^ aerosol 10%, Traumon^®^ gel 10% (both manufactured by MEDA Manufacturing GmbH, Cologne, Germany), Voltaren^®^ Max 2%, Voltaren^®^ Emulgel^®^ 1% (both manufactured by GSK Consumer Healthcare GmbH & Co. KG, Munich, Germany).

Strat-M^®^ membranes, 25% ammonia solution, acetonitrile isocratic grade, 2-propanol, phosphate-buffered saline pH 7.4, were from Merck (Darmstadt, Germany). Chloroacetic acid, secondary pharmaceutical standards of Diclofenac Sodium, Etofenamate, Ibuprofen, Ketoprofen, and Naproxen were purchased from Sigma-Aldrich (Darmstadt, Germany). Water was purified with a Milli-Q IQ plus system (Merck Millipore, Burlington, MA, USA).

### 2.2. PH, Conductivity, and Viscosity Measurements

The pH and conductivity measurements of all the studied products were performed using a SevenCompact Duo S213 pH-meter coupled with InLab Viscous Pro-ISM (for semisolids) or InLab Expert Pro-ISM (for solutions) and InLab 731-ISM electrodes (all from Mettler-Toledo GmbH, Greifensee, Switzerland), respectively. The electrodes were calibrated on certified buffer solutions pH 4.0, 7.0, and 9.0 and conductivity standards 147, 1413, and 12,880 µS/cm (all from Reagecon Diagnostics Ltd., Clare, Ireland). In addition, the viscosity of semi-solids was measured by an IKA Rotavisc Me-Vi viscometer (IKA-Werke GmbH & Co. KG, Staufen, Germany) coupled to spindle #SP-11 at 10 and 75 rpm. All measurements were conducted in quadruplicate at room temperature, and the results are shown in Table 1.

### 2.3. In Vitro Permeation Test (IVPT)

Twelve-hour in vitro permeation tests were performed using Strat-M^®^ membranes and Vision^®^ Microette^TM^ vertical diffusion cell (VDC, 7.0 mL, 1.767 cm^2^) automated test system (Hanson Research, Inc., Chatsworth, CA, USA). Phosphate-buffered saline pH 7.4, 6.6, or 5.8 was used as the receptor medium. About 0.55 mL of each semisolid formulation was applied to the Strat-M^®^ membrane in the donor compartment (Hanson VDC dosage wafer), and each cell was occluded with a glass disc to avoid evaporation. Precisely 300 µL of each liquid (aerosol) preparation was dosed to the Strat-M^®^ membrane in the threaded cell top for 7 mL VDC and closed with a screw cap. Samples were automatically collected from the receptor compartments at predefined time intervals (2, 4, 6, 9, 12 h) and replaced with the same amount of the fresh receptor medium.

There were two sets of IVPTs conditions used in this study. The first one was used to optimize IVPT conditions. DEA and ETF were studied in this part as model drugs with the same molecular mass (Table S1) but different physicochemical properties (logP, pKa, formulation pH and viscosity). The second one was based on the optimized conditions to compare NSAIDs diffusion from all tested products.

IVPTs conditions were optimized based on Plackett–Burman experimental design [17] to screen the effect of seven factors in an eight-run statistical model on the permeation rate (flux) of DEA and ETF from reference formulations (i.e., Voltaren^®^ Emulgel^®^ 1% and Traumon^®^ gel 10%, respectively). First, the following factors were evaluated: 2-propanol concentration in the receptor medium (10% vs. 40%) in order to verify if it can maintain the sink conditions, receptor medium temperature (32 °C vs. 37 °C), rotation speed (600 rpm vs. 1000 rpm), PBS pH (5.8 vs. 7.4) to evaluate the influence of medium pH on ETF and DEA fluxes, medium degassing (not degassed vs. degassed), stirring while sampling (unstirred vs. stirred), replacement medium volume (1 mL vs. 2 mL). One additional center point, where numeric factors were set midway between their low and high levels, was included in Plackett–Burman design to detect curvature in the response (i.e., the existence of second-order effects). All experiments were performed in triplicate at this stage. Next, the ratios of each Traumon^®^ gel sample’s slope to that of each Voltaren^®^ Emulgel^®^ sample’s slope (ETF/DEA flux ratios) were calculated and used for further statistical evaluations to establish the most discriminative and bio-predictive test parameters (based on etofenamate and diclofenac bioavailability data presented by Rannou et al. [1] we had expected the ETF/DEA ratio at least > 3). Thus, the obtained data were subjected to statistical analysis using Minitab^®^ 18.1 software (Minitab, Inc., State College, PA, USA) to analyze screening design. Then the optimized IVPT conditions were used to evaluate the NSAIDs diffusion from all the tested products. At this stage, all formulations were tested in six replicates.

To provide documented evidence that the functions of the VDCs comply with the USP specifications, the cell dimension evaluations, operational qualification (OQ), and performance qualification (PQ) were performed before and after the API permeation study. In addition, the temperature of the receptor medium, stirring speed, replacement/sampling procedure were continuously checked and recorded during every run.

The obtained permeation profiles were described employing linear regression equations according to Equation (1):

AQ/S = a√t + b,(1)

where AQ—the accumulated quantity of the drug in receptor medium [mg]; S—the surface of Strat-M^®^ membrane [cm^2^]; t—time [h]. The slope of the regression line (a) represented the rate of permeation of the drug (i.e., flux) from the product through Strat-M^®^ membrane per unit surface area (mg/cm^2^) versus square root of time (h^−2^). The permeability coefficient (K_p_) was calculated from Equation (2):

K_p_ = J_ss_/C_d_, (2)

where C_d_ is the initial concentration of the drug in the formulation applied on the membrane surface (mg/cm^3^), and J_ss_ is the steady-state flux (mg/cm^2^ h) (it comes from the permeation rate calculated from the Equation (1)).

### 2.4. UHPLC Analysis

The NSAIDs concentrations in the samples were analyzed immediately after IVPTs by a UHPLC method using Nexera-i LC- 2040C 3D Plus chromatograph coupled with a photodiode array detector, both operated by LabSolutions software v.6.82 (all from Shimadzu Co., Kyoto, Japan). Luna Omega Polar^®^ 1.6 µm C18 100 Å column 50 × 2.1 mm with SecurityGuard^TM^ Ultra Cartridge Fully Porous Polar C18 2.1 mm ID (all from Phenomenex^®^, Torrance, CA, USA) were used. The mobile phase was a mixture of 4 g/L chloroacetic acid pH 3.0 with acetonitrile (50:50, (*v*/*v*)) at a flow rate of 0.4 mL/min (isocratic conditions). The mobile phase was pre-filtered under vacuum through OlimPeak^TM^ 0.2 μm hydrophilic PTFE filter (Teknokroma, Barcelona, Spain) and degassed using an ultrasonic degasser (Sonorex Digiplus, Bandelin, Berlin, Germany) for 0.5 h. The autosampler, column oven, and detector temperatures were 4, 25, and 40 °C, respectively. The injection volumes, UV detection wavelengths, and retention times of NSAIDs are shown in Table S2.

This method was optimized first and then validated according to The International Council for Harmonization (ICH) guidelines [21], including specificity, linearity, range, accuracy, precision (repeatability and intermediate precision), the limit of detection (LOD), the limit of quantification (LOQ), robustness and stability of each API used in the study. Moreover, to ensure the quality of UHPLC determinations of API concentrations in the receptor media operational qualification (OQ), performance qualification (PQ), and performance verification (PV) tests of the analytical instrument were performed before and after the API permeation study.

### 2.5. Statistical Analysis

Exploratory data analyses were performed using MS Excel and DDSolver [22]. Minitab^®^ 18.1 software (Minitab, Inc., State College, PA, USA) was used to develop Plackett–Burman experimental design to screen independent variables. The statistical evaluation consisted of identifying statistically significant effects (*p* < 0.05) according to ANOVA and Pareto charts, evaluating the model’s fitting (R^2^ value and lack of fit test), and confirming the homoscedasticity and normal distribution residuals. In addition, to elucidate the relationship between the variables, response surface maps (RSM) were constructed. All results, unless stated otherwise, are expressed as mean ± standard deviation.

## 3. Results and Discussion

The physicochemical properties (molecular weight, log P, pKa, water solubility) and biological properties (IC50 (drug concentration that inhibits 50% of the enzyme activity) value for COX1 and COX2) of diclofenac (including diclofenac sodium and diethylamine), ketoprofen, naproxen (including sodium), ibuprofen and etofenamate are shown in Table S1.

### 3.1. Characterisation of NSAIDs Formulations

Table 1 presents the compositions and physicochemical characteristics of commercial topical products with NSAIDs.

Apart from one preparation (Dolgit^®^ cream), the rest of the studied semisolids are gels with carbomer as the gelling agent, except for one (Olfen^®^) gelled by cellulose derivatives. Both Voltaren^®^ formulations are emulsion type gels (emulgels), meaning oily droplets dispersed in an aqueous phase [23]. The optimum viscosity of carbomer gel is achieved at a pH of 5.0–7.0 [24]. The studied carbomer gels’ pH falls in the range of 5.15–7.48, being the highest for both Voltaren^®^ emulgels (7.41 and 7.45) and Naproxen EMO gel (7.48). Apart from the viscosity aspect, the pH of a topical preparation should be as close as possible to that of human skin, not to cause irritation or drying. From all of the studied semisolids, Traumon^®^ gel (5.15) and Dolgit^®^ cream (5.99) have the pH closest to physiological. Both aerosol formulations have pH close to neutral. Looking at the conductivity data of all the studied formulations, Naproxen EMO gel and Ketospray^®^ stand out, presenting much greater conductivity values than the rest of the preparations. It may result from high sodium hydroxide content in both products (pH values above 7).

Viscosity is an important physical property of topical formulations that may affect the rate of drug release and influences skin application (spreadability and skin feel). When the viscosity of a gel is too low, after applying to the skin, the gel has to be spread very fast as it quickly runs off. However, as far as the drug release is concerned, the literature data are discrepant if the increased viscosity improves or impedes the process [25]. From all the studied commercial formulations, Olfen^®^ gel was characterized by the lowest viscosity (at 10 rpm), typical for cellulose gels. The most outstanding viscosity was observed for both Traumon^®^ and Naproxen EMO gels. For all the semisolids, the viscosity was lower as the rotational speed of the viscometer increased. When the speed increases, the normally disarranged molecules of the vehicle are caused to align their long axes in the flow direction. Such orientation reduces the internal resistance of the material and hence decreases the viscosity making skin application easier [26]. We did not notice any correlation between the viscosities of semisolids and their permeation rates. Binder et al. concluded that the viscosity of hydrogel formulations seems to play a subordinate role in the skin penetration of an incorporated model drug [25].

### 3.2. Quantification of NSAIDs

A successful permeation test needs a reliable and precise analytical method to quantify the permeated drug in a receptor fluid. Ideally, the same method should enable quantifying all tested APIs under the same conditions. The UHPLC method is considered a quick and precise quantitative method for active substances in pharmaceutical products. It allows for the isolation of an analyzed substance from other product components. The use of a photodiode array (PDA) detector makes it possible to collect spectra for light-absorbing compounds in the 200–800 nm range with acceptable sensitivity and selectivity. After an extensive literature search, we have not found a single HPLC method applicable to quantify all five NSAIDs (DNa/DEA, ETF, IBU, KTP, NPX). Based on the literature review presented in Tables S3–S7, the chosen NSAIDs can be divided into two groups due to the mobile phase composition used in HPLC analysis. ETF and DEA/DNa can be determined using a mobile phase of acetonitrile, methanol, water, and a phosphate buffer at an appropriate pH with a defined ingredient ratio. For the determination of ETF, phosphate buffer (pH-adjusted to 6.0 with orthophosphoric acid) and methanol in the ratio of 20:80 % (*v*/*v*) were used as the mobile phase [27] (Table S6). For DNa and DEA determination (Table S3), the most straightforward mobile phase was a mixture of acetonitrile and methanol 70:30 % (*v*/*v*) [28] or methanol in water 30:70 % (*v*/*v*) [29], respectively. NPX, KTP, and IBU are the second group of APIs determined on the C18 HPLC column. However, except acetonitrile or methanol and water, acidifiers are present in the mobile phase: orthophosphoric acid, chloroacetic acid, sodium acetate, or a small amount of acetic acid (Tables S4–S6).

Thus we have decided to develop and validate a new method. The structure and size of selected API molecules gave the possibility of using the same or similar chromatographic conditions and, above all, the same column—C18 type.

The validation parameters of the analytical method used for the assay of APIs by UHPLC-UV were evaluated based on the analytical performance parameters such as specificity, linearity, precision, and accuracy. The results are shown in Table S8. The specificity of the method was ensured since none of the excipients used in the commercial formulations and receptor media interfered with particular API quantification. The linear regression analysis values revealed that the calibration curves fit the linear model with correlation coefficients ≥ 0.995. Besides, the low values of the standard errors of the slopes and intercepts were obtained. The methodology adopted to determine the NSAIDs concentrations had a high repeatability level and accuracy, as demonstrated by coefficients of variation below 2.5% and recovery levels between 98.7–100.8%. Those results are similar to the literature data concerning HPLC methods for NSAIDs quantification in IVPTs (cited in Supplementary Material Tables S3–S7). However, the values of LOD and LOQ seem to be higher as they were calculated using the calibration function to estimate the standard deviation (ICH approach) and the concentration ranges were adjusted to the needs of our permeation studies. It is worth noting that this assay has a short run time (retention times of all APIs are below 3 min) and reduced mobile phase solvent usage (due to the low flow rate 0.4 mL/min).

Thus an efficient, precise and fast chromatographic method was developed. It enabled to quantify all the APIs using the same column and mobile phase with different detection wavelengths only.

### 3.3. Optimization of IVPT Conditions

We performed optimization and validation of IVPT conditions using a Hanson’s vertical diffusion cell setup. Thus, the first stage of the study was focused on exploiting Plackett–Burman experimental design [17] to screen the effect of seven factors in eight runs with one additional center point (Table 2) on the permeation rate (flux) of DEA or ETF from commercial formulations for cutaneous application (Voltaren^®^ Emulgel^®^ 10 mg/g or Traumon^®^ gel 100 mg/g, respectively). We examined the influence of different test parameters (receptor-media composition, temperature and pH, media degassing and stirring speed (stirrer efficiency) as well as sampling volume and frequency on sink conditions) on the in vitro drug permeation profiles from semisolid preparations with model NSAIDs—DEA and ETF.

**Figure 1:**
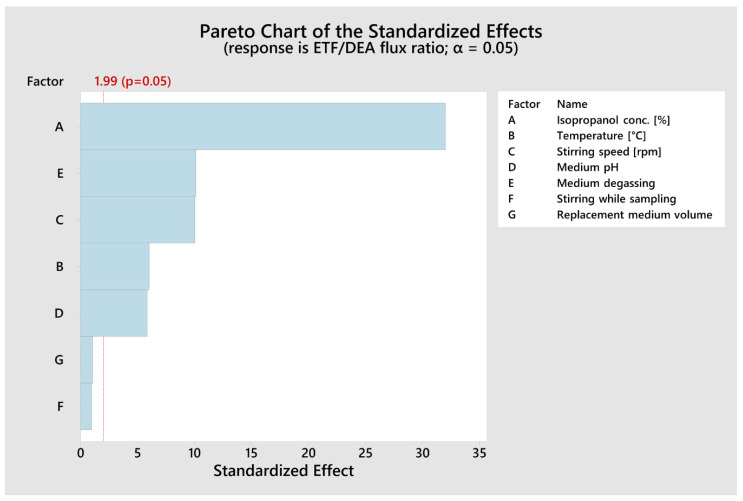
Pareto chart of the standardized effects of independent variables on ETF/DEA flux ratio values (α = 0.05). The red dotted line represents the lowest significant value of standardized effect at α = 0.05.

The statistical evaluation consisted of identifying statistically significant effects (*p* < 0.05) according to ANOVA (Table 3) and Pareto charts (Figure 1), evaluating the model’s fitting (R^2^ value and lack of fit test) and confirming the homoscedasticity and normal distribution residuals.

**Figure 2:**
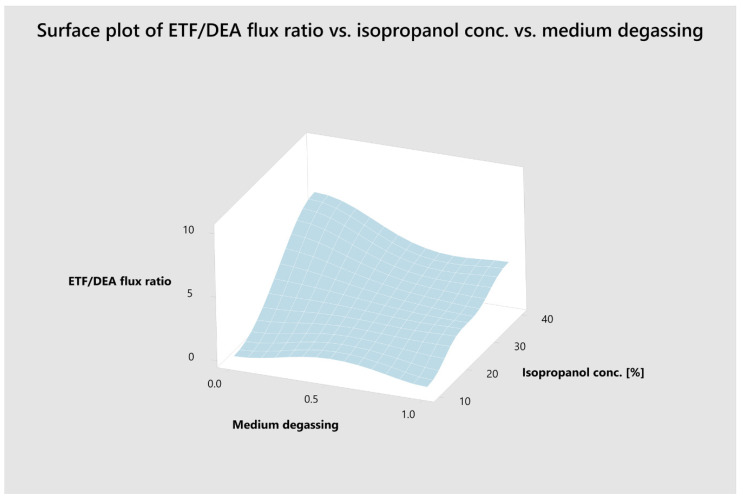
Response surface (3-D) plot illustrating the effect of isopropanol concentration and medium degassing on ETF/DEA flux ratio values.

To elucidate the relationship between the most important variables, response surface maps (RSM) were constructed (Figure 2).

The Plackett–Burman design recognizes the independent factors affecting the response variables and identifies the most significant factors. It revealed that isopropanol concentration, medium degassing, stirring speed, temperature and medium pH had a significant effect (*p* < 0.001, α = 0.05) on the ETF/DEA flux ratios (Table 2 and Table 3, Figure 1). The following mathematical model (R^2^ = 0.9478, R^2adj.^ = 0.9420) was established for ETF/DEA flux ratio value:

ETF/DEA flux ratio value = −8.07 + 0.16396 Isopropanol Conc. [%] + 0.1848 Temp. [°C] − 0.003851 Stirring Speed [rpm] + 0.5604 Medium pH − 1.553 Medium Degassing + 0.146 Stirring While Sampling + 0.162 Replacement Medium Volume + 0.069 Center Point(3)

Our results indicate that, from the seven factors studied, the major contributor to the NSAIDs’ permeation across Strat-M^®^ membrane is the composition of the receptor fluid, which is in good agreement with other studies [30,31]. Medium degassing and its stirring speed also have a significant impact on the process. An increase in alcohol concentration increased the rate and extent of ETF permeation (Table 2) due to the high solubilization power of the receptor fluid. Our additional research proved that diffusional sink conditions are maintained for DEA in each tested medium but for ETF only in the medium with 40% (*v*/*v*) of 2-propanol (data not shown), allowing sufficient drug permeation over a substantial time period. The sink conditions are an essential presupposition so that the drug concentration in the receptor medium does not limit the permeation rate. Precisely the increase of isopropanol concentration from 10% to 40% increased ETF permeation flux even up to 132 fold, while DEA flux up to 1.6 fold and the ETF/DEA flux ratio value up to 105 fold (Table 2). It also improved the discriminating power of the IVPT in accordance with in vivo studies of DEA and ETF absorption and bioavailability from topical formulations [1,32].

On the other hand, high concentration of isopropanol in the receptor medium may raise completely different concerns like the dissolution of lipids from the Strat-M^®^ membrane. Therefore, it can be presumed that the over-proportional increase in the ETF flux is not only due to the increase in its solubility, but it may be the sign of the dissolution of the membrane’s lipids. However, it is known that synthetic lipids in the Strat-M^®^ membrane are located mainly in the top layer [33], which is in contact with the tested formulation, and they are absent in the third layer, which is in contact with the receptor fluid [7,34]. Thus, the disruption of the barrier integrity is likely to be limited to the lipid-based top layer of the artificial membrane, as suggested by Arce et al. [33]. In our opinion, the extraction/dissolution of lipids from the Strat-M^®^ membrane depends mainly on the composition (presence of organic solvents) of the tested formulations. Traumon^®^ gel contains large amount of 2-propanol (confidential data) as a co-solvent, thanks to which ETF is dissolved in this product. Isopropanol could dissolve/extract the lipids in the apical side of the membrane, thus making ETF penetration easier [35,36] especially under sink conditions.

Medium outgassing decreases ETF/DEA flux ratios, probably due to isopropanol content reduction during ultrasounds and vacuum filtration treatment [37]. Similarly, an increase in stirring speed decreases the discriminative power of the test. However, to avoid hindered diffusion by the low liquid mixing speed in the receptor compartment, we had chosen the typical range of steering speed in Hanson’s vertical diffusion cell (i.e., 600–1000 rpm) when designing the IVPT [37,38]. Increasing the medium temperature resulted in higher values of ETF/DEA flux ratios, as it is suggested that the temperature increases the solvent power of the receptor fluid. Thomas et al. [39] demonstrated that heat application in conjunction with topically applied formulations could increase flux values.

**Figure 3:**
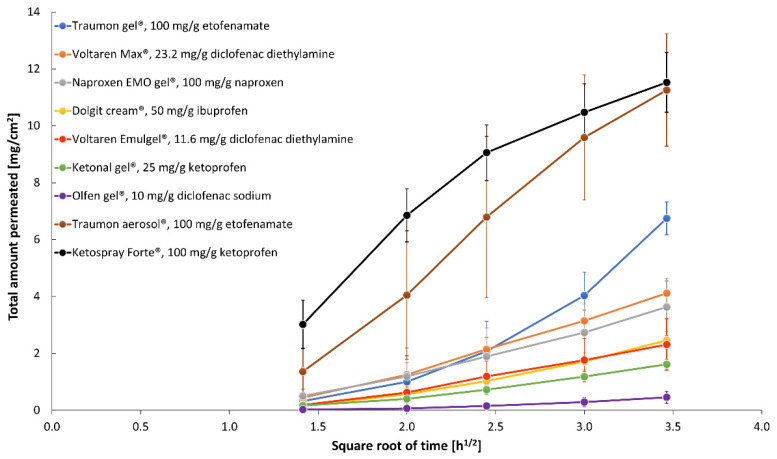
Permeation profiles (mean ± SD; *n* = 6) of NSAIDs from selected topical products across Strat-M^®^ membrane obtained in optimized in vitro test conditions.

**Figure 4:**
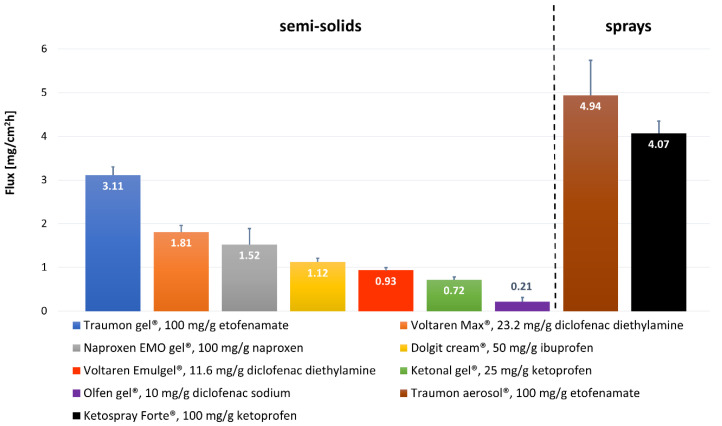
Comparison of NSAIDs permeation rates (mean ± SD; *n* = 6) from selected marketed topical formulations across Strat-M^®^ membrane obtained in optimized in vitro test conditions.

Our study proved that the Plackett–Burman design was an efficient tool to optimize key study parameters during IVPT method development, to identify conditions for a sensitive and discriminating IVPT study that can appropriately characterize the NSAIDs formulations. The following test parameters were regarded as being applicable for obtaining discriminative in vitro permeation profiles through Strat-M^®^ membrane from topical ETF and DEA reference formulations: the composition of the receptor fluid (40% (*v*/*v*) isopropanol and 60% (*v*/*v*) PBS pH 7.4) as the parameter of the most significant influence, not degassed receptor fluid with the temperature of 37 °C, a magnetic stirring bar with helix stirrer driven at 600 rpm. Thus, these optimized IVPT conditions were applied to evaluate NSAIDs’ in vitro permeation profiles (Figure 3) and parameters (Figure 4 and Table 4) from selected, marketed formulations.

### 3.4. Comparative Analysis of NSAIDs Permeation under Optimized In Vitro Conditions

There is little evidence in the literature showing that topical NSAIDs preparations can deliver therapeutic concentrations of drugs to underlying tissues. Undoubtedly the rate-limiting step of this transport is the drug partitioning into the stratum corneum, which is in part influenced by the relative solubility of the drug in the formulation and in the stratum corneum [40]. It is also clear that the absolute amount of the drug permeating the skin is strongly dependent upon the type of the vehicle (formulation) in which the API is applied—monophasic vs. multiphasic, vehicle viscosity, pH, presence of permeation-enhancing excipients [41]. Apart from the formulation properties, other critical factors influencing the flux across the healthy human skin include the drug’s concentration and physical state in the vehicle (solubilized vs. suspended), the drug’s molecular weight, salt vs. free acid or base form, etc. A proportional increase in the flux can be achieved by increasing the concentration of the dissolved drug. According to Fick’s law of diffusion, the excess drug in the formulation acts as a reservoir at a higher concentration above the solubility. It helps in maintaining constant flux for a prolonged period and thus increases the permeation. It is also known that greater flux is achieved when the drug is in solubilized rather than suspended form [42,43]. One cannot forget about the state of the skin that also plays an important role, as different skin diseases or topically applied drugs can significantly affect the permeation rate. The scope of our study did not include these parameters as they deserve a separate study. However, some general considerations according to the formulations’ ingredients have to be made.

Olfen^®^ gel (DNa) presents the slowest permeation rate, having at the same time the lowest NSAID concentration (along with Voltaren^®^ Emulgel^®^) from all the studied formulations (10 mg/g). Voltaren^®^ Emulgel^®^ (DEA) and Olfen^®^ (DNa) contain the same drug (but different salt form) in the same concentration. The first gel presents 4.4 times faster permeation rate and 4.4 times higher value of permeation coefficient (Figure 4, Table 4). Minghetti et al. [44] concluded that diclofenac salts had proved effective in promoting the drug’s permeation in vitro, provided that the salt-forming base is organic. A salt of this type offers a better partition coefficient and a higher activity coefficient, especially in water. The nature of the gelling agent and, even more importantly, the presence of penetration enhancers in the formulation may also influence the differences in permeation. Olfen^®^ (DNa) contains only isopropyl alcohol, while Voltaren^®^ Emulgel^®^ (DEA) includes isopropyl alcohol and propylene glycol as permeation enhancers. These components in vivo can diffuse to the skin surface and increase the permeation of drugs, either by disrupting the lipid structure of the stratum corneum or by increasing the solubility of the drug in the skin (i.e., increasing the partition coefficient of the drug between the skin and the vehicle) [45]. The study of Haltner-Ukomadu et al. [40] revealed opposite results to ours. They studied in vitro permeation of Olfen^®^ (DNa), Voltaren^®^ Emulgel^®^ (DEA), and Voltaren^®^ Max (DEA) gels to PBS pH = 7.4, achieving the greatest cumulative amount of diclofenac in the receptor fluid after 48 h for Olfen^®^ (DNa) gel (360.9 µg/cm^2^), while the lowest amount for Voltaren^®^ Emulgel^®^ (DEA) (122.6 µg/cm^2^). However it has to be emphasized that the authors used human ex vivo skin as the permeation membrane. Trying to explain the results they stated that “diclofenac in the hydrogel is immediately available for diffusion into the skin compared with the emulsion gel in which diclofenac must first release from the lipid phase to be available for penetration of the skin”. Interestingly Pradal et al. [23] published even more contrary results. They compared the permeation rates of two diclofenac salts to PBS with 5% BSA, revealing that diclofenac diethylamine 1.16% emulsion gel presented statistically significant higher permeation through human skin than diclofenac sodium 5% gel. It proves that higher concentration may not always lead to greater absorption through the skin. The authors claimed that the presence of emollients such as cocoyl caprylocaprate and paraffin in Voltaren^®^ Emulgel^®^ (DEA) could improve the level of skin hydration by occlusion, which favors drug absorption.

Voltaren^®^ Max (DEA) is a very similar preparation to Voltaren^®^ Emulgel^®^ (DEA) with diclofenac diethylamine in doubled concentration (2.32%). Its flux across the Strat-M^®^ membrane is almost twice as high as the flux of Voltaren^®^ Emulgel^®^, while the permeability coefficients are almost equal for those gels (Table 4). Considering the compositions of both Voltaren^®^ preparations, it can be noticed that Voltaren^®^ Max has the addition of a third penetration enhancer—oleyl alcohol. Long-chain fatty alcohols are effective penetration enhancers for a variety of drugs [46,47,48].

The fluxes of Naproxen EMO, Dolgit^®^, and Ketonal^®^ present decreasing order according to NSAIDs’ concentrations: 10% NPX, 5% IBU, 2.5% KTP; that seems to be the determining parameter here. However, after normalizing the permeation flux to the initial concentration of the drug in the donor compartment (*K_p_* values at Table 4 calculated according to Equation (2)) we obtain the reverse order to the drug dose. Although the drugs in those three gels are different, they are present in acidic form and have similar molecular masses, logP and pKa values (Table S1). Based on the literature review, we can conclude that NPX [49] and IBU [41,50] are suspended in their product vehicles, whereas KTP is fully dissolved [51]. They are weak acids, so at pH 7.4 (receptor fluid), they undergo an ionization process providing them good solubility. However, Chantasart et al. [52] revealed that the donor solution pH was a significant factor influencing skin permeation (they used human epidermal membrane) for the NSAIDs when the receiver’s pH was maintained at 7.4. They concluded that the NSAIDs’ apparent permeability coefficients increased when the donor solution’s pH decreased, consistent with the increase in the fraction of unionized NSAIDs in the donor solution at lower pH and the unionized free acids of NSAIDs as the main contributors to skin permeation.

The flux of ETF is definitely the highest from all the studied semisolids. Traumon^®^ (ETF) gel contains two penetration enhancers (isopropyl alcohol and propylene glycol), similarly to the two other formulations (Voltaren^®^ Max, Voltaren^®^ Emulgel^®^). So it is not the composition of the preparation that seems to have a profound impact on the permeation here, but the drug itself. Enhanced permeation of ETF could be attributed to its high concentration (the value of its *K_p_* is three times lower than that of DEA, Table 4). Pure ETF is a yellowish, highly viscous oil at room temperature. It has the highest logP value from all the studied NSAIDs (Table S1). ETF owes its physicochemical properties to its specific alcohol–ether–ester structure, and this structure gives this molecule its high lipophilicity [32]. Somewhat contrary results were obtained by Kopečná et al. [48]. The authors compared the permeation rates and skin retention of Voltaren^®^ Max (DEA) and four ETF gels (two 5% and two 10%; including Traumon^®^). Diclofenac emulgel delivered comparable amounts of API to Traumon^®^ gel. However, both the membrane used (human ex vivo skin) and the receptor fluid (phosphate-buffered saline at pH 7.4 with 5% bovine serum albumin) were different in that study comparing to ours. PBS with 5% BSA as the receptor phase is generally considered to imitate human serum and is recommended for in vitro tests of transdermal preparations where the APIs have a systemic effect [53]. Topical NSAIDs are not intended to be transdermals, but they are designed to penetrate the skin and accumulate in adjacent tissues in which they exert a local effect. Due to the extremely poor water solubility of ETF (Table S1), the choice of receptor fluid providing proper sink conditions for IVPT is crucial. The Plackett–Burman experimental design used in our optimization studies identified the amount of isopropanol in the receptor phase as the most significant factor influencing ETF permeation rates. That is why we used 40% isopropanol in PBS as the receptor phase. Marto et al. [24] used 40% of ethanol with PBS (pH = 7.4) as the receptor fluid to compare the permeation rates of ETF from hydroalcoholic gels.

From the two topical spray formulations tested (KTP and ETF in the same concentration) again, ETF has a higher flux. However, the cumulative amounts permeated at 12 h are almost equal for the two formulations. Both preparations have a few permeation enhancers: Ketospray^®^ forte (KTP) contains four (propylene glycol, isopropyl alcohol, soya lecithin, and ethanol) and Traumon^®^ aerosol (ETF) contains three (propylene glycol, isopropyl alcohol, and diisopropyl adipate). Fatty acid–alcohol esters like diisopropyl adipate have been shown to possess good solubilizing properties for NSAIDs and to enhance skin permeation of those drugs. However, they are more effective for enhancing the permeation of hydrophilic than lipophilic drugs. The enhancement effects of those diesters may be due to their causing lipid extraction in the skin [54]. A greater permeation rate for ETF may be explained by its higher lipophilicity, enabling the drug to easier cross the membrane. What is more, the pH effect may favor ETF here, as the difference between the pH of the formulation and the pKa of the drug is more significant for ketoprofen, suggesting its increased ionization that may impair membrane penetration.

Another aspect of the results that needs to be emphasized is the greater permeation rate of NSAIDs from aerosols than semisolids. We can hypothesize that it is a much lower viscosity in favor of those liquid formulations and a significant percentage of volatile solvents acting as permeation enhancers in aerosols. However, those results may not reflect in vivo effect. During our IVPTs of sprays, each cell was closed with a screw cap, so there was no possibility of evaporation of volatile excipients. However, it does happen post-application in real life conditions and can alter the composition and performance of these topically applied liquid formulations. It has been shown that evaporation and permeation of solvents can result in a saturated drug solution followed by its precipitation. The initial evaporation and saturation of solution accelerate drug delivery, but this advantage is lost once the drug precipitates [39].

The total permeated amounts (Q_12h_) for all the studied formulations are in accordance with their fluxes and range from 0.45 to 11.53 mg/cm^2^ (Table 4). Comparing to the literature data [26,40,43,44], those amounts appear to be relatively high. However, the receptor fluid chosen in our experiment (40% (*v*/*v*) isopropanol and 60% (*v*/*v*) PBS pH 7.4) and its temperature (37 °C) offer excellent solubilizing properties, and it may be responsible for those high values. Pradal [47] compared the permeation of IBU and diclofenac from different topical formulations using human skin. Cumulative absorption to PBS with 5% BSA as a receptor fluid ranged from 119 to 25282 ng/cm^2^ after 24 h. Applying the drugs at single finite doses, the author wanted to mimic ‘in-use’ conditions and that is why a maximum flux was not reached in all formulations within the 24 h testing window. Ibuprofen permeated to a greater extent than diclofenac. However, its higher concentrations (5% and 10%) compared to diclofenac (1% and 2%) in the tested formulations must also contribute to the result [47]. Sanna et al. [26] achieved the cumulative amount of diclofenac permeated at 1.5 h from Voltaren^®^ Emulgel^®^ to PBS (pH = 7.4) in the range 244.3–258.5 µg/cm^2^ depending on the membrane used. Sacha et al. [43] used PBS (pH 7.4)/methanol (60:40 % (*v*/*v*)) at 32 °C as the receptor phase to compare the in vitro release rates of three diclofenac gels achieving rates 691–825 μg/cm^2^ after 3 h.

The analgesic and anti-inflammatory actions of NSAIDs are, to a considerable extent, dose-dependent. Therefore, within the dose limits that can be delivered topically, the greater the percentage of skin permeation, the better the clinical response [41]. Topical NSAIDs produce high drug concentrations in the dermis, muscle, synovium, and joint cartilage, while plasma drug concentrations are less than 10% of those obtained after oral administration [55].

Etofenamate levels were reported 10- to even 1000-fold higher in fasciae, muscles and the periosteum than in plasma after cutaneous application [42]. The bioavailability of 5% ETF following topical application is high (>20%) compared with 1% diclofenac—around 6%, and 1–7% for other topical NSAIDs [1]. The results are in accordance with ours since the flux for ETF is around 3.3 times higher than Voltaren^®^ Emulgel^®^ (DEA). This may be explained by the high lipophilicity of ETF (logP value around 5) [56]. The drug was specifically designed to meet topical anti-inflammatory treatment requirements, such as adequate anti-inflammatory and analgesic efficacy, good local and systemic tolerability, and good transcutaneous penetrating ability [32]. It is rapidly metabolized to flufenamic acid in vivo, which has similar properties as the parent drug [57].

## 4. Conclusions

An efficient and fast UHPLC method was developed, optimized, and validated for simultaneous determination of all the studied NSAIDs. It may come in handy for other researchers dealing with NSAIDs.

During IVPT method development, we employed Plackett–Burman’s design as a powerful tool to optimize discriminating parameters that could appropriately characterize the NSAIDs formulations. As a result, the following test parameters were regarded as being applicable for obtaining discriminative in vitro permeation profiles through STRAT-M^®^ membrane from semisolid ETF and DEA reference topical formulations: the composition of the receptor fluid (40% (*v*/*v*) isopropanol and 60% (*v*/*v*) PBS pH 7.4) as the parameter of the greatest influence, not degassed receptor fluid with the temperature of 37 °C, a magnetic stirring bar with helix stirrer driven at 600 rpm. Those parameters may be applicable for similar studies, possibly with other NSAIDs.

Due to the optimized IVPT method we managed to compare in equal conditions different drugs from the same group (NSAID) present in different topical preparations formulated with different excipients. What is more, the utilization of Strat-M^®^ membrane makes the study reproducible and provides the possibility of repeating the same conditions by other laboratory.

ETF permeation rate from Traumon^®^ gel across STRAT-M^®^ membrane was 1.7–14.8 times faster than other NSAIDs from the rest of the tested semisolids but 1.6 times slower than the ETF from liquid formulation (Traumon^®^ aerosol).

We can hypothesize that the results may partially reflect the degree of APIs in vivo absorption to the site of inflammation and indicate its effectiveness. What is more excipients comparison along with the permeation results may be the useful source of practical information when formulating topical NSAIDs preparations.

Some literature data comparing Strat-M^®^ membrane with human skin ex vivo have been available so far [7,12,13]. However such a comparison with different NSAIDs would be highly useful and may be regarded as a future work that needs to be done to extend our knowledge about the correlation of permeation results between artificial membranes and human ex vivo skin.

## Figures and Tables

**Table 1:** Characteristics of commercial topical products with NSAIDs.

Commercial Name and Batch Number (Type of Formulation)	Drug and Its Concentration (mg/g)	Excipients (from Manufacturer’s Label)	pH Mean ± SD (*n* = 4)	Conductivity (mS/m) Mean ± SD (*n* = 4)	Viscosity (mPa·s) Mean ± SD (*n* = 4)	
					10 rpm	75 rpm
Dolgit^®^, Lot: 811007, (cream)	Ibuprofen (IBU), 50	Methyl 4-hydroxybenzoate sodium, medium-chain triglycerides, glycerol monostearate, polyoxyethylene 30 stearate, polyoxyethylene 100 stearate, propylene glycol, xanthan gum, lavender oil, orange oil, purified water	5.99 ± 0.05	15.57 ± 0.18	32,567 ± 1097	7397 ± 55
Ketonal^®^, Lot: JE4531, (gel)	Ketoprofen (KTP), 25	Carbomer, triethanolamine, lavender essential oil, ethanol 96%, purified water	6.31 ± 0.08	210.45 ± 2.90	54,367 ± 1801	11,311 ± 328
Naproxen EMO, Lot: 81671, (gel)	Naproxen (NPX), 100	Chloralhydrate, levomenthol, ethanol 96%, ethyl *p*-hydroxybenzoate, sodium hydroxide, carbomer, purified water	7.48 ± 0.08	1049.50 ± 4.95	62,233 ± 1290	12,851 ± 311
Olfen^®^, Lot: T26318A, (gel)	Diclofenac sodium (DNa), 10	Isopropyl adipinate, lactic acid, isopropyl alcohol, sodium pyrosulphite, hydroxypropylcellulose, hydroxyethylcellulose, purified water	6.13 ± 0.02	63.28 ± 0.62	26,733 ± 2003	6933 ± 127
Traumon^®^, Lot: 3832641, (gel)	Etofenamate (ETF), 100	Carbomer, fatty alcohol-polyglycol-ether, sodium hydroxide, isopropyl alcohol, macrogol 400, propylene glycol, purified water	5.15 ± 0.05	10.90 ± 0.02	62,567 ± 1060	13,005 ± 100
Voltaren^®^ Emulgel^®^, Lot: 2G3R, (emulsion gel)	Diclofenac diethylamine (DEA), 11.6 (equivalent to 10 mg/g diclofenac sodium)	Carbomer, cocoyl caprylocaprate, diethylamine, isopropyl alcohol, liquid paraffin, macrogol cetostearyl ether, perfume, propylene glycol, purified water	7.41 ± 0.04	140.10 ± 0.57	30,633 ± 513	7623 ± 110
Voltaren^®^ Max, Lot: 876D, (emulsion gel)	Diclofenac diethylamine (DEA), 23.2 (equivalent to 20 mg/g diclofenac sodium)	Butylhydroxytoluene, carbomer, cocoyl caprylocaprate, diethylamine, isopropyl alcohol, liquid paraffin, macrogol cetostearyl ether, oleyl alcohol, perfume, propylene glycol, purified water	7.45 ± 0.02	195.40 ± 1.56	30,167 ± 153	6670 ± 437
Ketospray^®^ forte, Lot: 216554-21, (topical spray, solution)	Ketoprofen (KTP), 100	Propylene glycol, isopropyl alcohol, soya lecithin, ethanol, sodium dihydrogen phosphate dihydrate, disodium phosphate dodecahydrate, sodium hydroxide, peppermint oil, purified water	7.22 ± 0.02	806.15 ± 1.77	n/a	n/a
Traumon^®^ aerosol, Lot: 3823121, (topical spray, solution)	Etofenamate (ETF), 100	Diisopropyl adipate, fatty alcohol-polyglycol-ether, macrogol 400, isopropyl alcohol, propylene glycol, purified water	6.45 ± 0.13	1.66 ± 0.01	n/a	n/a

**Table 2:** Experimental plan of Plackett–Burman seven-factor eight-run screening design with a center point (C) and the observed response values (DEA and ETF fluxes and their ratio).

Exp. No.	Factors with Levels							Responses Mean ± SD (*n* = 3)		Fluxes Comparison
	Isopropanol Conc. (%)	Temp. (°C)	Stirring Speed (rpm)	Medium pH	Medium Degassing	Stirring While Sampling	Replacement Medium Volume (mL)	ETF Flux (µg/cm^2^h)	DEA Flux (µg/cm^2^h)	ETF/DEA Flux Ratio Value
1	10	32	600	7.4	yes	yes	1	30 ± 5	416 ± 40	0.07 ± 0.01
2	10	32	1000	7.4	no	no	2	53 ± 7	518 ± 43	0.10 ± 0.01
3	10	37	600	5.8	yes	no	2	50 ± 4	436 ± 72	0.12 ± 0.02
4	10	37	1000	5.8	no	yes	1	59 ± 3	518 ± 12	0.11 ± 0.01
5 (C)	25	34.5	800	6.6	no/yes ^a^	no/yes ^b^	1.5	1014 ± 164	528 ± 32	1.92 ± 0.29
6	40	32	600	5.8	no	yes	2	3976 ± 256	690 ± 76	5.82 ± 0.64
7	40	32	1000	5.8	yes	no	1	1614 ± 56	674 ± 63	2.41 ± 0.21
8	40	37	600	7.4	no	no	1	3015 ± 688	414 ± 42	7.32 ± 1.58
9	40	37	1000	7.4	yes	yes	2	2824 ± 630	623 ± 18	4.54 ± 0.88

^a^ half of the volume of receptor medium degassed, ^b^ for the time of sampling, the stirrer was turned off and then restarted again when half of the sample volume was collected.

**Table 3:** Summary of analysis of variance (ANOVA) of Plackett–Burman screening design batches for ETF/DEA flux ratios.

Source	DF	Adj. SS	Adj. MS	*F*-Value	*p*-Value Prob > F
Model	8	555.532	69.441	163.44	**<0.001**
Linear	7	552.311	78.902	185.70	**<0.001**
Isopropanol Conc. (%)	1	435.510	435.510	1025.01	**<0.001**
Temperature (°C)	1	15.368	15.368	36.17	**<0.001**
Stirring Speed (rpm)	1	42.711	42.711	100.52	**<0.001**
Medium pH	1	14.470	14.470	34.06	**<0.001**
Medium Degassing	1	43.394	43.394	102.13	**<0.001**
Stirring While Sampling	1	0.384	0.384	0.90	0.345
Replacement Medium Volume (mL)	1	0.474	0.474	1.12	0.294
Curvature	1	0.031	0.031	0.07	0.788
Error	72	30.592	0.425		
Total	80	586.123			

DF—Total Degrees of Freedom; Adj. SS—Adjusted Sum of Squares; Adj. MS—Adjusted Mean Squares; The F-value is the test statistic used to determine whether the term is associated with the response; The *p*-value is a probability that measures the evidence against the null hypothesis. Lower probabilities provide stronger evidence against the null hypothesis. 95% significant values are given in bold.

**Table 4:** NSAID permeation parameters from selected commercial formulations through Strat-M^®^ membrane in optimized in vitro test conditions.

Commercial Formulation (API)	Drug Flux (*J_ss_*) (mg/cm^2^h)	Determination Coefficient (*R*^2^)	Permeability Coefficient (*K_P_*) (cm/h)	Total Amount Permeated at 12 h (*AQ*_12 h_) (mg/cm^2^)
Dolgit^®^ cream 5% (IBU)	1.12 ± 0.09	0.9735 ± 0.0095	(2.24± 0.18) × 10^−2^	2.45 ± 0.31
Ketonal^®^ gel 2.5% (KTP)	0.72 ± 0.06	0.9767 ± 0.0093	(2.88 ± 0.25) × 10^−2^	1.61 ± 0.18
Naproxen EMO gel 10% (NPX)	1.52 ± 0.36	0.9878 ± 0.0070	(1.52 ± 0.36) × 10^−2^	3.63 ± 0.85
Olfen^®^ gel 1% (DNa)	0.21 ± 0.10	0.9272 ± 0.0309	(2.11 ± 0.98) × 10^−2^	0.45 ± 0.22
Traumon^®^ gel 10% (ETF)	3.11 ± 0.19	0.9110 ± 0.0532	(3.11 ± 0.19) × 10^−2^	6.75 ± 0.58
Voltaren^®^ Emulgel^®^ 1.16% (DEA)	0.93 ± 0.06	0.9887 ± 0.0053	(9.32 ± 0.61) × 10^−2^	2.31 ± 0.91
Voltaren^®^ Max 2.32% (DEA)	1.81 ± 0.15	0.9921 ± 0.0079	(9.05 ± 0.75) × 10^−2^	4.12 ± 0.43
Ketospray ^®^ 10% (KTP)	4.07 ± 0.28	0.9378 ± 0.0343	(4.07 ± 0.28) × 10^−2^	11.53 ± 1.05
Traumon^®^ aerosol 10% (ETF)	4.94 ± 0.80	0.9463 ± 0.0428	(4.94 ± 0.80) × 10^−2^	11.26 ± 1.97

## Data Availability

The data presented in this study are available through whole manuscript and supplementary material.

## Supplementary Materials

The following are available online at https://www.mdpi.com/article/10.3390/pharmaceutics13081305/s1, Table S1: The selected physicochemical and biological properties of selected NSAIDs. Table S2: UHPLC method parameters: sample injection volumes, retention times, and NSAIDs analytical wavelengths. Table S3: The permeation test results of topical diclofenac medications and HPLC methods for its quantification in receptor fluids. Table S4: The permeation test results of topical ketoprofen medications and HPLC methods for its quantification in receptor fluids. Table S5: The permeation test results of topical naproxen medications and HPLC methods for its quantification in receptor fluids. Table S6: The permeation test results of topical ibuprofen medications and HPLC methods for its quantification in receptor fluids. Table S7: The permeation test results of topical etofenamate medications and HPLC methods for its quantification in receptor fluids. Table S8: The validation parameters for the assays of NSAIDs by the UHPLC-UV method.
