# Supplementary material for: Optimization and Evaluation of the In Vitro Permeation Parameters of Topical Products with Non-Steroidal Anti-Inflammatory Drugs through Strat-M® Membrane
Source: Pharmaceutics. 2021 Aug 20;13(8):1305. doi: 10.3390/pharmaceutics13081305 (PMC8398299; doi:10.3390/pharmaceutics13081305)
Supplement: Supplementary file 1 [file pharmaceutics-13-01305-s001.zip › pharmaceutics-1301675-supplementary.pdf]

pharmaceutics

MDPI

Supplementary Materials: Optimization and evaluation of the *in vitro* permeation parameters of topical products with non-steroidal anti-inflammatory drugs through Strat-M® membrane

Bartłomiej Milanowski, Hanna Wosicka-Frąckowiak, Eliza Główka, Małgorzata Sosnowska, Stanisław Woźny, Filip Stachowiak, Angelika Suchenek and Dariusz Wilkowski

Table S1. The selected physicochemical and biological properties of the studied NSAIDs.

Active pharmaceutical ingredient (API)	Structure / formula	Molar mass [g·mol⁻¹]	Log P (experimental; octanol / water)	pK_a at 25°C	Solubility in water at 25ºC [mg L^{-1}]	IC 50 (COX-2) [µmol L^{-1}]
Diclofenac	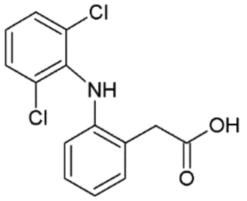 $C_{14}H_{11}Cl_2NO_2$	296.1	4.0 [1]<br>4.4 [2-5]<br>4.2 [6]<br>4.5 [7-9]	3.80 [3,4,10]<br>4.18 [11]<br>4.0 [12]<br>4.50 [2]	17.8 [1,10]<br>3.5 [12,13]	COX-1: 0.5, 0.3; COX-2: 0.038, 0.002 [14];<br>COX-2: 0.003 ± 0.001 [15]
Diclofenac sodium	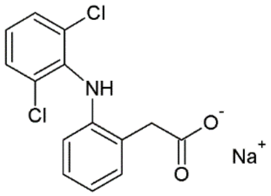 $C_{14}H_{10}Cl_2NO_2Na$	318.1	0.7 [7,16]		19 100 [17]<br>32 400 (37ºC) [18]	
Diclofenac diethylamine	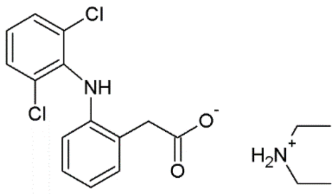 $C_{18}H_{22}Cl_2N_2O_2$	369.3	0.853 (octanol/buffer pH=7.4) [19]		4 062.3 [10]<br>12 925.5 (20 ºC) [10,20]	

Ketoprofen	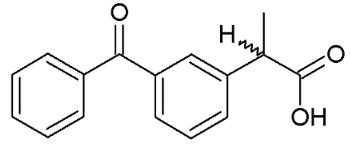 $C_{16}H_{14}O_3$	254.3	3.12 [2,4]<br>1.93 [21]<br>3.2 [22]	4.5 [23]<br>4.6 [2,4]<br>4.39, 4.45, 4.76 [22]	210 [24]<br>880 (37ºC) [23]<br>10 (22-24 ºC) [22,25]<br>253 (37ºC) [22]	COX-1: 61, 5.;<br>COX-2: 2.9, 0.24 [14];<br>COX-2: 0.74 ± 0.37 [15]
Naproxen	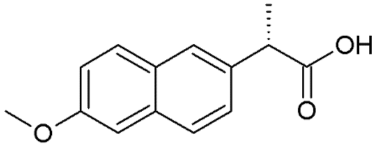 $C_{14}H_{14}O_3$	230.3	3.18 [4,25]<br>2.16 [21]<br>3.34 [2]	4.2 [23,26]<br>4.6 [4]<br>4.15 [2]	20 [24]<br>65.4 [27]<br>17 (ambient temperature) [28]<br>25 [29]	COX-1: 3.0, 3.8;<br>COX-2: 28, 35 [14]
Naproxen sodium	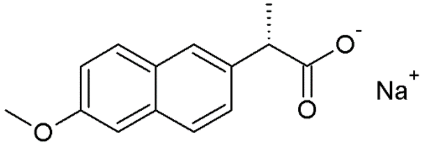 $C_{14}H_{13}O_3Na$	252.1			181 200 (22.9 ºC) [30]<br>200 000 [31]	
Ibuprofen	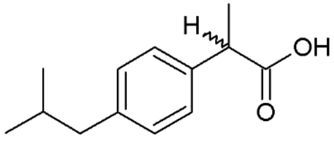 $C_{13}H_{18}O_2$	206.3	3.50 [2,4]<br>2.41 [21]	5.3 [23]<br>4.55 [4]<br>5.20 [2]	100 [23]	COX-1: 0.9, 2.6;<br>COX-2: 7.2, 20 [14]
Etofenamate	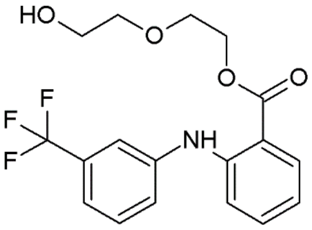 $C_{18}H_{18}F_3NO_4$	369.3	about 5 [32]	pKa1=6; pKa2=7 [33]<br>Flufenamic acid: about 4 [32]	Etofenamate is sparingly soluble in aqueous buffers (water solubility = 9.36 mg/L based on information at https://www.drugbank.ca/drugs/DB08984). For maximum solubility in aqueous buffers, etofenamate should be dissolved in alcohol and then diluted with the aqueous buffer of choice. Etofenamate has a solubility of approximately 100 mg/L in a 1:5 solution of ethanol : PBS (pH=7.2) using this method (https://www.caymanchem.com/pdfs/23674.pdf).	Flufenamic acid:<br>COX-1: 3.1;<br>COX-2: 9.3 [14]

Table S2. UHPLC method parameters: sample injection volumes, retention times, and NSAIDs analytical wavelengths.

**API**	**Injection volume [µL]**	**Retention time [min]**	**Analytical wavelength [nm]**
Diclofenac sodium	1.0	1.9	276
Etofenamate	0.5 (gel and spray)	2.8	286
Ibuprofen	2.0	2.0	229
Ketoprofen	1.0 (gel) and 0.5 (spray)	1.0	227
Naproxen	2.0	1.5	330

Table S3. The permeation test results of topical diclofenac medications and HPLC methods for its quantification in receptor fluids.

API	Formulation	Penetration studies	HPLC assay	References
Diclofenac sodium	Carbopol® 940 gel,<br><br>o/w microemulsion, Voltaren Emulgel	Franz diffusion cells<br>PS: 1.76 cm²; MT: Swiss albino rats skin;<br>RF: 20 mL of phosphate buffer (pH 7.4) ; TEMP: 37°C<br>SW: 1 g; RFA: 0.7 mL; TT: 8h; 600 rpm	---	[17]
Diclofenac epolamine	microemulsion, Poloxamer Microemulsion based gel, Flector® gel	Franz diffusion cells<br>PS: 4.9 cm²; MT: rat skin; RF: 300 mL phosphate buffer (pH 7.4) ; TEMP: 37°C;<br>SW: 1.0 g (1.3%); RFA: 4.0 mL; TT: 8h; 600 rpm	---<br>AW: UV 276 nm	[34]
Diclofenac sodium	laminated adhesive diclofenac containing patches (including capsaicin and hyoscyamine) or only with capsaicin and hyoscyamine	Franz diffusion cells<br>PS: 10.0 cm²; MT: rat skin; RF: PEG 300 solution in water (1:4); TEMP: 37°C; SW: ---; RFA: 0.6 mL; TT: 24 or 48h<br><br>0.1 ml of IS solution was added to the samples before injection onto the column	Glass column Separon SGX C-18 (150_3.3 mm I.D., 7 mm) (Tessek, Prague).<br>MP: methanol:phosphate buffer pH 6.5 (110:100) with an addition of tetrabutylammonium iodide (5 mM:l), FR: 0.5 ml/min, AW: 284 nm<br>RT: ~3.5 min	[35]
Ionized and nonionized diclofenac	suspension or solutions in McIlvaine buffer/ethanol (0-40%)	Franz diffusion cells<br>PS: 0.785 cm²; MT: rat skin; RF: 3 mL phosphate buffer (pH 7.2) (10-fold of solubility); TEMP: 37°C; SV: 3 mL; RFA: 0.02 mL; TT: 8h	HPLC apparatus (Model 655, Hitachi Ltd) equipped with a variable wavelength UV monitor.<br>Column: YMC Packed A-302 S-5 120A ODS 4.6 X 1.50 mm (Yamamura Chemical Laboratories Co., Ltd).<br>MP: 0.1% aqueous phosphoric acid-methanol (1: 4 v/v), FR: 1.0 ml/min, AW: 283 nm	[36]
Diclofenac potassium	tablets	---	Phenomenex LUNA C18 (25 cm x 4.6 mm i.d., 5 μ) column MP: acetonitrile: sodium dihydrogen orthophosphate (adjusted to pH 3.5 using orthophosphoric acid) in the ratio of 70:30 v/v,<br>FR: 1.0 mL/min, AW: 278 nm, RT: 9.4 min	[37]
Diclofenac sodium	propylene glycol, water, ethanol (saturated or unsaturated solution)	PAMPA plate<br>PS: 280μl cm²; MT: Strat-M™; RF: 280μl Krebs–Ringer bicarbonate buffer spiked with dextrose (Sigma–Aldrich, St. Louis, MO) and bovine serum albumin (4.5%, w/v; Fisher Scientific, Fair Lawn, NJ) ; TEMP: 37°C; SV: --- ; RFA: 0.02 mL; TT: 5h	---	[38]
Diclofenac sodium	suppositories	---	Lichrospher 100 RP 18 (5 μm, 25 × 4 cm; Merck). MP: methanol and phosphate buffer pH 2.5 (76:24, v/v),<br>FR: 1.0 mL/min, AW: 254 nm, RT: ~7 min	[39]
Diclofenac sodium	tablets	---	Zorbax SB n C 18 column 150 ◊ 4.6 mm<br>MP: methanol and water (60:40, v/v), FR: 1 mL/min, AW: 248 nm, RT: 2.73 min	[40]
Diclofenac	---	---	C-18 analytical reversed phase column (250mm×4.6mm i.d., 5μM particle size, Beckman, Palo Alto, CA, USA) MP: acetonitrile: sodium acetate buffer (75 mM, pH adjusted to 5.0 with acetic acid) in a ratio of 2:1.5 (v/v), FR: 0.5 ml/min, AW: 280 nm, RT: 16.2 min	[41]

Diclofenac diethylamine	nanoemulsion gel	---	Hypersil BDS-5 mm, RP-C8 column (250mm×4.6 mm, Thermo Scientific), MP: methanol and water (70:30 v/v) pH = 3.0±0.5 FR: 1 mL/min, AW: 280 nm, RT: 9.5 min	[42]

PS – Penetration Surface; MT – Membrane Type; RF – receptor Fluid; SW – Sample Weight; SV – Sample Volume; RFA – receptor Fluid Aliquot; TT – Total Time; MP – Mobile Phase; FR - Flow Rate; AW – Analytical Wavelength; RT – Retention Time

Table S4. The permeation test results of topical ketoprofen medications and HPLC methods for its quantification in receptor fluids.

API	Formulation	Penetration studies	HPLC assay	References
Ketoprofen	adhesive TDDs (transdermal drug delivery system)	Modified Franz diffusion cells<br>PS: 0.951 cm²; MT: cellophane membrane; RF: phosphate buffer (pH 7.4); TEMP: 32°C; SV: ---; RFA: 1.0 mL; TT: 7h;	---	[43]
Ketoprofen	isopropyl myristate solution	---	Phenomenex Luna C18 5µm column (250x4.6 mm) with a Luna 5-pm guard Column (30x4.6 mm)<br>MP: acetonitrile-methanol-water (36:54:10, v/v/v),<br>FR: 1.2 mL/min, RT: 2.3 min, AW: 265 nm	[44]
Ketoprofen	sodium alginate gel (2.5%)	Franz diffusion cells<br>PS: 2.5 cm²; MT: regenerated cellulose, Strat-M™; RF: phosphate buffer (pH 5.6 and 7.4) 125.0 mL; TEMP: 37°C; SW: 5.0 g; RFA: ---; TT: 24h;	---	[45]
Ketoprofen	KP-TDDS	Franz diffusion cells<br>PS: 1.7679 cm²; MT: full thickness rat skin; RF: phosphate buffer (pH 7.4) 12.0 mL; TEMP: 32°C; SW: ---; RFA: 500 µl; TT: 30h;	Nucleosil 100–5 C18 column (5 µm, 250x4.6mm)<br>temp. = 40°C, MP: acetonitrile/pH 3.5 phosphate buffer mixture (55:45 v/v), FR: 1.0 ml/min,<br>RT: 2.73 min, AW: 254 nm	[46]
Ketoprofen	benzyl alcohol / ethanol / Solutol®HS 15 / water nanoemulsion	Franz diffusion cells<br>PS: 1.77 cm²; MT: mouse skin; RF: phosphate buffer (pH 7.4) 11.5 mL; TEMP: 37°C; SV: 2 mL; RFA: 0.2 mL; TT: 24h;	ODS column (Tosoh, Tskgel ODS-80TS, 4.6 mm i.d × 150 mm)<br>MP: acetonitrile and pH 7.4 phosphate buffer (78:22 v/v),<br>FR: 1 mL/min, RT: ---, AW: 258 nm	[47]
Ketoprofen	Cremophor RH40-PEG 400 microemulsions	Franz diffusion cells<br>PS: 2.31 cm²; MT: shed snake skin; RF: phosphate buffer (pH 7.4) PBS 6.0 mL; TEMP: 32°C; SV: ---; RFA: 1.0 mL; TT: 12h	C18 column (Waters, USA, particle size = 5µm; column dimension = 4.6 × 150 mm)<br>FR: 1 ml/min, MP: 0.1% (v/v) phosphoric acid: methanol (25:75, v/v), AW: 254 nm	[48]

PS – Penetration Surface; MT – Membrane Type; RF – Receptor Fluid; SW – Sample Weight; SV – sample volume; RFA – Receptor Fluid Aliquot; TT – Total Time; MP – Mobile Phase; FR - Flow Rate; AW – Analytical Wavelength; RT – Retention Time

Table S5. The permeation test results of topical naproxen medications and HPLC methods for its quantification in receptor fluids.

API	Formulation	Penetration studies	HPLC assay	References
Naproxen	solution and commercial gels: Reuxen, Tecnifar, Naprosyn	Franz diffusion cells<br>PS: 1.0 cm^2; MT: cellulose acetate and polyethersulphon; RF: phosphate buffer (pH 7.4) PBS 4.0 mL; TEMP: 32°C; SW: 1 g; RFA: 0.1-1.0 mL; TT: 24h	---	[49]
Naproxen sodium	tablets	---	RP -UFLC<br>C18G column (250 × 4.6mm i.d., 5µm)<br>MP: methanol:10mM tetra butyl ammonium hydrogen sulfate (80:20, v/v), FR: 1.2mL/min, RT: 4.5 min, AW: 231 nm	[50]
Naproxen	microemulsions	Franz diffusion cells<br>PS: 3.4618 cm^2; MT: cellulose membrane; RF: phosphate buffer (pH 7.4) 25.0 mL; TEMP: 32°C; SW: 5 g; RFA: 2.0 mL; TT: 24h	---<br>AW: 271 nm	[51]
Naproxen	solid lipid nanoparticles	Franz diffusion cells<br>PS: 3.4618 cm^2; MT: rat skin; RF: 50:50 ethanol/water mixture (5.5 mL); TEMP: 37°C; SV: 10 mL; RFA: ---; TT: 24h	Agilent Eclipse XDB-C18 column (5µm, 4.6 × 250 mm)<br>MP: 40:20:40 acetonitrile, methanol and acetic acid (1% v/v), FR: 0.7 ml/min, RT: 11 min, AW: 230 nm	[52]
Naproxen	o/w microemulsions	Franz diffusion cells<br>PS: 0.75 cm^2; MT: cellulose membrane; RF: 50:50 ethanol/water mixture (4.5 mL); TEMP: 35°C; SV: 500 µl; RFA: 200 µl; TT: 22h	Waters Symmetry, 4.6 × 15 cm reverse phase column (C18).<br>MP: methanol/phosphate buffer pH 2.5 (65:35 v/v), FR: 1 ml/min, AW: 274 nm, RT: ---	[53]

PS – Penetration Surface; MT – Membrane Type; RF – Receptor Fluid; SW – Sample Weight; SV – sample volume; RFA – Receptor Fluid Aliquot; TT – Total Time; MP – Mobile Phase; FR - Flow Rate; AW – Analytical Wavelength; RT – Retention Time

Table S6. The permeation test results of topical ibuprofen medications and HPLC methods for its quantification in receptor fluids.

API	Formulation	Penetration studies	HPLC assay	References
Ibuprofen	methanol solution	---	Rainin Microsorb 3µm; 10 × 4.6 mm reverse phase column (C18)<br>MP: acetonitrile, 0.1 M sodium acetate (35:65 v/v; glacial acetic acid added to obtain pH=6.4), FR: 1.5 ml/min<br>AW: 220 nm, RT: 4.0 min, Temp.: 50°C	[54]
Ibuprofen	ointment	---	Precolumn: Pye Unicam RP-18, 10µm; 4.6 × 30 mm<br>Analytical column: Pye Unicam RP-18, 10µm; 4.6 × 100 mm<br>MP: THF, 0.2M NaH_2PO_4 (45:55, v/v; pH=4), FR: 1.0 ml/min, AW: 219 nm, RT: 6.6 min, Temp.: -	[55]
Ibuprofen	gel	Franz diffusion cells<br>PS: 1.131 cm^2; MT: synthetic hydrophilic GH Polypro filter, pore size: 0.45nm; RF: phosphate buffer, pH=7.4 (6.5 mL); TEMP: 32°C; S: 1g; RFA: 500 µl; TT: 8h	C18 column, 5µm; 3.9 × 150 mm<br>MP: acetonitrile, 0.01M H_3PO_4 (45:55, v/v), FR: 1.0 ml/min, AW: 264 nm, RT: 7.129 min, Temp.: -	[56]
Ibuprofen	gel	Franz diffusion cells<br>PS: 1.00 cm^2; MT: excised porcine abdominal skin; RF: phosphate buffer, pH=7.4 (8.0 mL); TEMP: 32.0±0.5°C; S: -;RFA: 200 µl; TT: 8h	HyperClone reverse phase C18 column, 5µm; 4.6 × 250 mm<br>MP: acetonitrile, phosphate buffer pH=7.4 (gradient; 30:70, 10 min), FR: 1.0 ml/min, AW: 233 nm, RT: -, Temp.: -	[57]
Ibuprofen	gel	Vertical diffusion cells<br>PS: about 2 cm^2; MT: silicone membrane (Silatos 120 µm thickness), pig ear skin; RF: phosphate-buffered saline pH=7.4 67mM (6.5 mL); TEMP: 37°C; S: -; RFA: 200 µl; TT: 8h	Merck Lichrospher100 RP18, 5µm<br>MP: acetonitrile, citrate buffer pH=2.4 (55:45 v/v), FR: 1.2 ml/min, AW: 227 nm<br>RT: about 5 min, Temp.: -	[58]
Ibuprofen	microemulsion	Franz diffusion cells<br>PS: 2.8 cm^2; MT: porcine ear skin; RF: phosphate-buffered saline pH=7.4 (7.0 mL); TEMP: 37°C; S: 1.0 g; RFA: 500 µl; TT: 8h	Merck Lichrospher C18, 5µm; 4. 6 × 250 mm<br>MP: acetonitrile, sodium acetate buffer pH=2.5 (70:30 v/v), FR: 0.8 ml/min, AW: 264 nm, RT: about 7.5 min, Temp.: -	[59]
Ibuprofen, acetaminophen, chlorzoxazone	tablets	---	Kromasil C8, 5µm; 4. 6 × 250 mm<br>MP: acetonitrile, 0.2% trimethylamine solution (pH=3.2 obtained with H_3PO_4) (50:50 v/v), FR: 1.5 ml/min, AW: 215 nm, RT: about 12 min, Temp.: 20±1°C	[60]
Ibuprofen	spray, gel, cream, foam	Franz diffusion cells<br>PS: 1.15 cm^2; MT: human skin; RF: phosphate-buffered saline pH=7.4, ethanol (75:25, v/v) (2.5 mL); TEMP: 37.0±0.5°C; S: 5mg/cm^2; RFA: 200 µl; TT: 48h	RP Apex ODS column; 4. 6 × 250 mm<br>MP: acetonitrile, buffer (60:40, pH=2.5), FR: 1.5 ml/min, AW: 225 nm, RT: -, Temp.: -	[61]
Ibuprofen	gel	Franz diffusion cells<br>PS: 0.64 cm^2; MT: hairless mouse skin; RF: isotonic phosphate buffer pH=7.2 with 0.1% (v/v) formaldehyde and 0.5% (w/v) Brij 58 (5.1 mL); TEMP: 37.0±0.5°C; S: 200 µl; RFA: 300 µl; TT: 24h	Reverse phase C18 column, 5µm; 4. 6 × 150 mm<br>MP: acetonitrile, monobasic sodium phosphate buffer pH=3 (60:40), FR: 1.0 ml/min, AW: 254 nm, RT: 3.8 min, Temp.: -	[62]

PS – Penetration Surface; MT – Membrane Type; RF – Receptor Fluid; S – Sample Amount; RFA – Receptor Fluid Aliquot; TT – Total Time; MP – Mobile Phase; FR - Flow Rate; AW – Analytical Wavelength; RT – Retention Time

Table S7. The permeation test results of topical etofenamate medications and HPLC methods for its quantification in receptor fluids.

API	Formulation	Penetration studies	HPLC assay	References
Etofenamate	solution in mobile phase	---	Qualisil Gold C18 column, 5µm; 4.6 × 250 mm<br>MP: methanol, 0.2% triethylamine in water (v/v) at 85:15 ratio (v/v) (pH adjusted to 6.5 with 10% v/v H_3PO_4), FR: 1.2 ml/min, AW: 286 nm, RT: 5.3 min, Temp.: -	[33]
Etofenamate	drug solutions	---	Qualisil BDS C18 column, 5µm; 4.6 × 250 mm<br>MP: methanol, phosphate buffer (80:20, v/v) (pH adjusted to 6.0 with H_3PO_4), FR: 1.0 ml/min, AW: 286 nm, RT: 7.5 min, Temp.: -	[63]
Etofenamate	plasma, synovial fluid and tissue samples	---	Nucleosil 7 C18 column, 5µm; 4.0 × 250 mm<br>MP: -, FR: 1.0 ml/min, AW: 286 nm, RT: about 15 min, Temp.: -	[64]
Etofenamate	gel	Franz diffusion cells<br>PS: 1 cm^2; MT: hydrophilic polysulfone filters, human abdominal skin; RF: ethanol, phosphate buffer pH=7.4 (2:3, v/v); TEMP: 32±2°C; SW: 0.2±0.1g; RFA: 200 µl; TT: 12h	Reversed phase Lichrospher 100 RP18 column, 5µm; 4.0 × 125 mm<br>MP: methanol, acetonitrile, water (45:35:20, v/v/v), FR: 1.0 ml/min, AW: 286 nm, RT: -, Temp.: -	[65]

PS – Penetration Surface; MT – Membrane Type; RF – Receptor Fluid; SW – Sample Weight; RFA – Receptor Fluid Aliquot; TT – Total Time; MP – Mobile Phase;
FR - Flow Rate; AW – Analytical Wavelength; RT – Retention Time

Table S8. The validation parameters for the assays of NSAIDs by the UHPLC-UV method.

**Parameter**	Diclofenac sodium	Etofenamate	Ibuprofen	Ketoprofen	Naproxen
Specificity[a]	✓	✓	✓	✓	✓
Linearity range[b] [mg/mL]	0.22-1.00	0.83-4.11	0.20-0.65	0.12-0.43	0.35-1.78
Slope	5253215	2960365	1803474	9451898	1753264
Intercept	27620	-207535	96769	21058	17550
Correlation coefficient (r)	0.997	0.998	0.995	0.995	0.999
SE of slope	106529	55011	49399	249923	11159
SE of intercept	70805	149930	21420	70864	13151
LOD [mg/mL]	0.07	0.28	0.06	0.04	0.04
LOQ [mg/mL]	0.22	0.83	0.20	0.12	0.12
Stability of API in standard solution[c]	>12h	>12h	>12h	>12h	>12h
System suitability[d] [%]	0.28	0.30	0.21	0.21	0.21
Interday precision[e] [%]	0.39	1.75	1.10	1.74	0.50
Intraday precision[f] [%]	0.90	2.44	1.27	2.28	1.24
Accuracy (recovery)[g] [%]	100.4±3.8	100.8±3.9	98.7±7.0	99.7±5.2	99.86±2.01

[a] the medium and placebo must not interfere with the determination of active substances,

[b] 5 relative concentrations of the solution (25 – 125% for each API, y=ax+b, r≥0,995, slope (a) – significant, intercept (b) – irrelevant),

[c] the relative difference in peak surface area values for the standard and test solution immediately after preparation and after "t" from preparation should not be greater than 2% (RSD ≤ 2%),

[d] based on 6 injections of standard solution with concentration corresponding to 100% of API (RSD standard for n=6 ≤ 2%),

[e] based on analysis of 6 samples of standard solution with concentration corresponding to 100% of API from 1 Vertical Diffusion Cell (RSD for one group of results ≤ 5%),

[f] based on 2 groups of results done by 2 analysts, where one group of results is based on analysis of 6 samples of standard solution with concentration corresponding to 100% of API from 1 Vertical Diffusion Cell by 1 analyst (RSD for one group of results ≤ 2%, the relative difference between means ≤ 5%, coefficient F calc.< F crit. (F crit.=5.05)),

[g] 5 relative concentrations of the solution (25 – 125% for each API, the average recovery value must be in the range of 95 - 105%, each result must fall within the specified confidence interval (average recovery value ± 2SD)).

References

1. Fini, A.; Fazio, G.; Gonzalez-Rodriguez, M.; Cavallari, C.; Passerini, N.; Rodriguez, L. Formation of ion-pairs in aqueous solutions of diclofenac salts. *Int J Pharm* **1999**, *187*, 163-173, doi:10.1016/s0378-5173(99)00180-5.
2. Barbato, F.; La Rotonda, M.I.; Quaglia, F. Interactions of non-steroidal anti-inflammatory drugs with phospholipids: comparison between octanol/buffer partition coefficients and chromatographic indexes on immobilized artificial membranes. *J Pharm Sci* **1997**, *86*, 225-229, doi:10.1021/js960233h.
3. Chuasuwan, B.; Binjesoh, V.; Polli, J.E.; Zhang, H.; Amidon, G.L.; Junginger, H.E.; Midha, K.K.; Shah, V.P.; Stavchansky, S.; Dressman, J.B., et al. Biowaiver monographs for immediate release solid oral dosage forms: diclofenac sodium and diclofenac potassium. *J Pharm Sci* **2009**, *98*, 1206-1219, doi:10.1002/jps.21525.
4. Fini, A. Solubility and solubilization properties of non-steroidal anti-inflammatory drugs. *International Journal of Pharmaceutics* **1995**, *126*, 95-102, doi:10.1016/0378-5173(95)04102-8.
5. Hallare, A.V.; Kohler, H.R.; Triebskorn, R. Developmental toxicity and stress protein responses in zebrafish embryos after exposure to diclofenac and its solvent, DMSO. *Chemosphere* **2004**, *56*, 659-666, doi:10.1016/j.chemosphere.2004.04.007.
6. Sintov, A.C. Cumulative evidence of the low reliability of frozen/thawed pig skin as a model for in vitro percutaneous permeation testing. *Eur J Pharm Sci* **2017**, *102*, 261-263, doi:10.1016/j.ejps.2017.03.024.
7. Balon, K.; Riebesehl, B.U.; Muller, B.W. Determination of liposome partitioning of ionizable drugs by titration. *J Pharm Sci* **1999**, *88*, 802-806, doi:10.1021/js9804213.
8. Ghosh, P.; Lee, D.; Kim, K.B.; Stinchcomb, A.L. Optimization of naltrexone diclofenac codrugs for sustained drug delivery across microneedle-treated skin. *Pharm Res* **2014**, *31*, 148-159, doi:10.1007/s11095-013-1147-8.
9. Rubbens, J.; Mols, R.; Brouwers, J.; Augustijns, P. Exploring gastric drug absorption in fasted and fed state rats. *Int J Pharm* **2018**, *548*, 636-641, doi:10.1016/j.ijpharm.2018.07.017.
10. O'Connor, K.M.; Corrigan, O.I. Comparison of the physicochemical properties of the N-(2-hydroxyethyl) pyrrolidine, diethylamine and sodium salt forms of diclofenac. *Int J Pharm* **2001**, *222*, 281-293, doi:S0378517301007177 [pii].
11. Andreeta, A.; Verde, C.; Babusci, M.; Muller, R.; Simpson, M.I.; Landoni, M.F. Comparison of Diclofenac Diethylamine Permeation Across Horse Skin from Five Commercial Medical Human Formulations. *Journal of Equine Veterinary Science* **2011**, *31*, 502-505, doi:10.1016/j.jevs.2011.03.011.
12. Cordero, J.A.; Alarcon, L.; Escribano, E.; Obach, R.; Domenech, J. A comparative study of the transdermal penetration of a series of non-steroidal anti-inflammatory drugs. *J Pharm Sci* **1997**, *86*, 503-508, doi:10.1021/js950346l.
13. Hadgraft, J.; du Plessis, J.; Goosen, C. The selection of non-steroidal anti-inflammatory agents for dermal delivery. *Int J Pharm* **2000**, *207*, 31-37, doi:10.1016/s0378-5173(00)00517-2.
14. Warner, T.D.; Giuliano, F.; Vojnovic, I.; Bukasa, A.; Mitchell, J.A.; Vane, J.R. Nonsteroid drug selectivities for cyclo-oxygenase-1 rather than cyclo-oxygenase-2 are associated with human gastrointestinal toxicity: A full in vitro analysis. *Proceedings of the National Academy of Sciences* **1999**, *96*, 7563-7568, doi:10.1073/pnas.96.13.7563.
15. Cordero, J.A.; Camacho, M.; Obach, R.; Domenech, J.; Vila, L. In vitro based index of topical anti-inflammatory activity to compare a series of NSAIDs. *European Journal of Pharmaceutics and Biopharmaceutics* **2001**, *51*, 135-142, doi:https://doi.org/10.1016/S0939-6411(00)00149-1.
16. Nii, T.; Ishii, F. Encapsulation efficiency of water-soluble and insoluble drugs in liposomes prepared by the microencapsulation vesicle method. *Int J Pharm* **2005**, *298*, 198-205, doi:10.1016/j.ijpharm.2005.04.029.
17. Sarigullu Ozguney, I.; Yesim Karasulu, H.; Kantarci, G.; Sozer, S.; Guneri, T.; Ertan, G. Transdermal delivery of diclofenac sodium through rat skin from various formulations. *AAPS PharmSciTech* **2006**, *7*, 88, doi:10.1208/pt070488.
18. Okumura, M.; Sugibayashi, K.; Ogawa, K.; Morimoto, Y. Skin permeability of water-soluble drugs. *Chem Pharm Bull (Tokyo)* **1989**, *37*, 1404-1406, doi:10.1248/cpb.37.1404.
19. Arora, P.; Mukherjee, B. Design, development, physicochemical, and in vitro and in vivo evaluation of transdermal patches containing diclofenac diethylammonium salt. *J Pharm Sci* **2002**, *91*, 2076-2089, doi:10.1002/jps.10200.
20. Kriwet, K.; Müller-Goymann, C.C. Diclofenac release from phospholipid drug systems and permeation through excised human stratum corneum. *International Journal of Pharmaceutics* **1995**, *125*, 231-242, doi:10.1016/0378-5173(95)00130-b.
21. Mura, P. Interactions of ketoprofen and ibuprofen with β-cyclodextrins in solution and in the solid state. *International Journal of Pharmaceutics* **1998**, *166*, 189-203, doi:10.1016/s0378-5173(98)00035-0.
22. Shohin, I.E.; Kulinich, J.I.; Ramenskaya, G.V.; Abrahamsson, B.; Kopp, S.; Langguth, P.; Polli, J.E.; Shah, V.P.; Groot, D.W.; Barends, D.M., et al. Biowaiver monographs for immediate-release solid oral dosage forms: ketoprofen. *J Pharm Sci* **2012**, *101*, 3593-3603, doi:10.1002/jps.23233.

23. Yiyun, C.; Tongwen, X. Dendrimers as potential drug carriers. Part I. Solubilization of non-steroidal anti-inflammatory drugs in the presence of polyamidoamine dendrimers. *Eur J Med Chem* **2005**, *40*, 1188-1192, doi:10.1016/j.ejmech.2005.06.010.
24. Ahmed, I.S.; Nafadi, M.M.; Fatahalla, F.A. Formulation of a fast-dissolving ketoprofen tablet using freeze-drying in blisters technique. *Drug Dev Ind Pharm* **2006**, *32*, 437-442, doi:10.1080/03639040500528913.
25. Meza, C.P.; Santos, M.A.; Romañach, R.J. Quantitation of drug content in a low dosage formulation by transmission near infrared spectroscopy. *AAPS PharmSciTech* **2017**, *7*, E206-E214, doi:10.1208/pt070129.
26. Bhise, K.S.; Dhumal, R.S.; Paradkar, A.R.; Kadam, S.S. Effect of drying methods on swelling, erosion and drug release from chitosan-naproxen sodium complexes. *AAPS PharmSciTech* **2008**, *9*, 1-12, doi:10.1208/s12249-007-9001-0.
27. Pacheco, D.P.; Martínez, F. Thermodynamic analysis of the solubility of naproxen in ethanol + water cosolvent mixtures. *Physics and Chemistry of Liquids* **2007**, *45*, 581-595, doi:10.1080/00319100701313862.
28. Tiong, N.; Elkordy, A.A. Effects of liquisolid formulations on dissolution of naproxen. *Eur J Pharm Biopharm* **2009**, *73*, 373-384, doi:10.1016/j.ejpb.2009.08.002.
29. Mura, P.; Faucci, M.T.; Bettinetti, G.P. The influence of polyvinylpyrrolidone on naproxen complexation with hydroxypropyl-β-cyclodextrin. *European Journal of Pharmaceutical Sciences* **2001**, *13*, 187-194, doi:10.1016/s0928-0987(01)00093-8.
30. Chavez, K.J.; Rousseau, R.W. Solubility and Pseudopolymorphic Transitions in Mixed Solvents: Sodium Naproxen in Methanol−Water and Ethanol−Water Solutions. *Crystal Growth & Design* **2010**, *10*, 3802-3807, doi:10.1021/cg1006956.
31. Kim, Y.S.; Mendez del Rio, J.R.; Rousseau, R.W. Solubility and prediction of the heat of solution of sodium naproxen in aqueous solutions. *J Pharm Sci* **2005**, *94*, 1941-1948, doi:10.1002/jps.20391.
32. Graham, G.G. Fenamates. In *Compendium of Inflammatory Diseases*, Parnham, M.J., Ed. Springer Basel: Basel, 2016; 10.1007/978-3-7643-8550-7_24pp. 477-482.
33. Peraman, R.; Bhadraya, K.; Reddy, Y.P.; Reddy, C.S.; Lokesh, T. Analytical Quality by Design Approach in RP-HPLC Method Development for the Assay of Etofenamate in Dosage Forms. *Indian J Pharm Sci* **2015**, *77*, 751-757, doi:10.4103/0250-474x.174971.
34. Fouad, S.A.; Basalious, E.B.; El-Nabarawi, M.A.; Tayel, S.A. Microemulsion and poloxamer microemulsion-based gel for sustained transdermal delivery of diclofenac epolamine using in-skin drug depot: in vitro/in vivo evaluation. *Int J Pharm* **2013**, *453*, 569-578, doi:10.1016/j.ijpharm.2013.06.009.
35. Klimes, J.; Sochor, J.; Dolezal, P.; Korner, J. HPLC evaluation of diclofenac in transdermal therapeutic preparations. *Int J Pharm* **2001**, *217*, 153-160, doi:10.1016/s0378-5173(01)00594-4.
36. Obata, Y.; Takayama, K.; Maitani, Y.; Machida, Y.; Nagai, T. Effect of ethanol on skin permeation of nonionized and ionized diclofenac. *International Journal of Pharmaceutics* **1993**, *89*, 191-198, doi:10.1016/0378-5173(93)90243-9.
37. Gowramma, B.; Rajan, S.; Muralidharan, S.; Meyyanathan, S.N.; Suresh, B. A validated RP-HPLC method for simultaneous estimation of paracetamol and diclofenac potassium in pharmaceutical formulation. *International Journal of ChemTech Research* **2010**, *2*, 676-680.
38. Karadzovska, D.; Riviere, J.E. Assessing vehicle effects on skin absorption using artificial membrane assays. *European Journal of Pharmaceutical Sciences* **2013**, *50*, 569-576, doi:https://doi.org/10.1016/j.ejps.2013.02.020.
39. Sznitowska, M.; Stokrocka, M. Determination of diclofenac released from suppositories using UV spectrophotometry, spectra derivative spectrophotometry and HPLC. *Acta Pol Pharm* **2007**, *64*, 401-405.
40. Kasperek, R. Determination of diclofenac sodium and papaverine hydrochloride in tablets by HPLC method. *Acta Pol Pharm* **2008**, *65*, 403-408.
41. Kaphalia, L.; Kaphalia, B.S.; Kumar, S.; Kanz, M.F.; Treinen-Moslen, M. Efficient high performance liquid chromatograph/ultraviolet method for determination of diclofenac and 4'-hydroxydiclofenac in rat serum. *J Chromatogr B Analyt Technol Biomed Life Sci* **2006**, *830*, 231-237, doi:10.1016/j.jchromb.2005.10.045.
42. Hamed, R.; Basil, M.; AlBaraghthi, T.; Sunoqrot, S.; Tarawneh, O. Nanoemulsion-based gel formulation of diclofenac diethylamine: design, optimization, rheological behavior and in vitro diffusion studies. *Pharm Dev Technol* **2016**, *21*, 980-989, doi:10.3109/10837450.2015.1086372.
43. Wongpayapkul, L.; Leesawat, P.; Rittirod, T.; Klangtrakul, K.; Pongpaibul, Y. Adhesive Property, in vitro Release and Permeation Studies of Ketoprofen Transdermal Drug Delivery Systems. *CMU. Jurnal* **2005**, *4*, 305-314.
44. Proniuk, S.; Lerkpulsawad, S.; Blanchard, J. A simplified and rapid high-performance liquid chromatographic assay for ketoprofen in isopropyl myristate. *J Chromatogr Sci* **1998**, *36*, 495-498, doi:10.1093/chromsci/36.10.495.
45. Salamanca, C.H.; Barrera-Ocampo, A.; Lasso, J.C.; Camacho, N.; Yarce, C.J. Franz Diffusion Cell Approach for Pre-Formulation Characterisation of Ketoprofen Semi-Solid Dosage Forms. *Pharmaceutics* **2018**, *10*, doi:10.3390/pharmaceutics10030148.

46. Wongpayapkul, L.; Leesawat, P.; Rittirod, T.; Klangtrakul, K.; Pongpaibul, Y. Effect of Single and Combined Permeation Enhancers on the Skin Permeation of Ketoprofen Transdermal Drug Delivery Systems. *CMU. Journal* **2006**, *5*, 41-52.
47. Kim, B.S.; Won, M.; Lee, K.M.; Kim, C.S. In vitro permeation studies of nanoemulsions containing ketoprofen as a model drug. *Drug Deliv* **2008**, *15*, 465-469, doi:10.1080/10717540802328599.
48. Ngawhirunpat, T.; Worachun, N.; Opanasopit, P.; Rojanarata, T.; Panomsuk, S. Cremophor RH40-PEG 400 microemulsions as transdermal drug delivery carrier for ketoprofen. *Pharm Dev Technol* **2013**, *18*, 798-803, doi:10.3109/10837450.2011.627871.
49. Farinha, A.; Toscano, C.; Campos, R.; Bica, A.; Hadgraft, J. Permeation of naproxen from saturated solutions and commercial formulations through synthetic membranes. *Drug Dev Ind Pharm* **2003**, *29*, 489-494, doi:10.1081/ddc-120018383.
50. Panda, S.S.; Bera, V.R.K.; Kumari, A. Ultrafast Liquid Chromatographic Determination of Naproxen Sodium in Pharmaceutical Dosage Form. *Columbia Int. Publ. Am. J. Mod. Chromatogr.* **2015**, *2*, 16–25.
51. Moghimipour, E.; Salimi, A.; Eftekhari, S. Design and characterization of microemulsion systems for naproxen. *Adv Pharm Bull* **2013**, *3*, 63-71, doi:10.5681/apb.2013.011.
52. Akbari, J.; Saeedi, M.; Morteza-Semnani, K.; Rostamkalaei, S.S.; Asadi, M.; Asare-Addo, K.; Nokhodchi, A. The design of naproxen solid lipid nanoparticles to target skin layers. *Colloids Surf B Biointerfaces* **2016**, *145*, 626-633, doi:10.1016/j.colsurfb.2016.05.064.
53. Montenegro, L.; Carbone, C.; Condorelli, G.; Drago, R.; Puglisi, G. Effect of oil phase lipophilicity on in vitro drug release from o/w microemulsions with low surfactant content. *Drug Dev Ind Pharm* **2006**, *32*, 539-548, doi:10.1080/03639040600599806.
54. Aravind, M.K.; Miceli, J.N.; Kauffman, R.E. Determination of ibuprofen by high-performance liquid chromatography. *J Chromatogr* **1984**, *308*, 350-353, doi:10.1016/s0021-9673(01)87566-x.
55. Haikala, V.E.; Heimonen, I.K.; Vuorela, H.J. Determination of ibuprofen in ointments by reversed-phase liquid chromatography. *J Pharm Sci* **1991**, *80*, 456-458, doi:10.1002/jps.2600800512.
56. Rasool, B.K.A.; Abu-Gharbieh, E.; Fahmy, S.; Saad, H.; Khan, S. Development and evaluation of ibuprofen transdermal gel formulations. *Trop J Pharm Res* **2010**, *9*, 355-363, doi:10.4314/tjpr.v9i4.58928.
57. Abioye, A.O.; Issah, S.; Kola-Mustapha, A.T. Ex vivo skin permeation and retention studies on chitosan-ibuprofen-gellan ternary nanogel prepared by in situ ionic gelation technique--a tool for controlled transdermal delivery of ibuprofen. *Int J Pharm* **2015**, *490*, 112-130, doi:10.1016/j.ijpharm.2015.05.030.
58. Herkenne, C.; Naik, A.; Kalia, Y.N.; Hadgraft, J.; Guy, R.H. Ibuprofen transport into and through skin from topical formulations: in vitro-in vivo comparison. *J Invest Dermatol* **2007**, *127*, 135-142, doi:10.1038/sj.jid.5700491.
59. Chen, H.; Chang, X.; Du, D.; Li, J.; Xu, H.; Yang, X. Microemulsion-based hydrogel formulation of ibuprofen for topical delivery. *Int J Pharm* **2006**, *315*, 52-58, doi:10.1016/j.ijpharm.2006.02.015.
60. Ravisankar, S.; Vasudevan, M.; Gandhimathi, M.; Suresh, B. Reversed-phase HPLC method for the estimation of acetaminophen, ibuprofen and chlorzoxazone in formulations. *Talanta* **1998**, *46*, 1577-1581, doi:10.1016/s0039-9140(98)00043-5.
61. Hadgraft, J.; Whitefield, M.; Rosher, P.H. Skin penetration of topical formulations of ibuprofen 5%: an in vitro comparative study. *Skin pharmacology and applied skin physiology* **2003**, *16*, 137-142, doi:10.1159/000069759.
62. Godwin, D.A.; Wiley, C.J.; Felton, L.A. Using cyclodextrin complexation to enhance secondary photoprotection of topically applied ibuprofen. *Eur J Pharm Biopharm* **2006**, *62*, 85-93, doi:10.1016/j.ejpb.2005.06.002.
63. Peraman, R.; Nayakanti, D.; Dugga, H.H.; Kodikonda, S. Development and Validation of a Stability-Indicating Assay of Etofenamate by RP-HPLC and Characterization of Degradation Products. *Sci Pharm* **2013**, *81*, 1017-1028, doi:10.3797/scipharm.1305-19.
64. Dannhardt, G.; Laufer, S.; Lehr, M. HPLC determination of etofenamate and flufenamic acid in biological material. *Clin Chem* **1988**, *34*, 2580-2581.
65. Marto, J.; Baltazar, D.; Duarte, A.; Fernandes, A.; Gouveia, L.; Militao, M.; Salgado, A.; Simoes, S.; Oliveira, E.; Ribeiro, H.M. Topical gels of etofenamate: in vitro and in vivo evaluation. *Pharm Dev Technol* **2015**, *20*, 710-715, doi:10.3109/10837450.2014.915571.
